# Triglyceride and Triglyceride-Rich Lipoproteins in Atherosclerosis

**DOI:** 10.3389/fmolb.2022.909151

**Published:** 2022-05-25

**Authors:** Bai-Hui Zhang, Fan Yin, Ya-Nan Qiao, Shou-Dong Guo

**Affiliations:** Institute of Lipid Metabolism and Atherosclerosis, Innovative Drug Research Centre, School of Pharmacy, Weifang Medical University, Weifang, China

**Keywords:** hypertriglyceridemia, cardiovascular disease, triglyceride-rich lipoprotein, residual risk, lipid-lowering

## Abstract

Cardiovascular disease (CVD) is still the leading cause of death globally, and atherosclerosis is the main pathological basis of CVDs. Low-density lipoprotein cholesterol (LDL-C) is a strong causal factor of atherosclerosis. However, the first-line lipid-lowering drugs, statins, only reduce approximately 30% of the CVD risk. Of note, atherosclerotic CVD (ASCVD) cannot be eliminated in a great number of patients even their LDL-C levels meet the recommended clinical goals. Previously, whether the elevated plasma level of triglyceride is causally associated with ASCVD has been controversial. Recent genetic and epidemiological studies have demonstrated that triglyceride and triglyceride-rich lipoprotein (TGRL) are the main causal risk factors of the residual ASCVD. TGRLs and their metabolites can promote atherosclerosis via modulating inflammation, oxidative stress, and formation of foam cells. In this article, we will make a short review of TG and TGRL metabolism, display evidence of association between TG and ASCVD, summarize the atherogenic factors of TGRLs and their metabolites, and discuss the current findings and advances in TG-lowering therapies. This review provides information useful for the researchers in the field of CVD as well as for pharmacologists and clinicians.

## Introduction

According to the WHO report in 2021, cardiovascular disease (CVD) is still the leading cause of death worldwide, and atherosclerotic CVD (ASCVD) is the most representative and dangerous one (Lin et al., 2021; WHO, 2021). Cholesterol carried by lipoproteins in blood is the major inducer of ASCVD. Among the lipoproteins, low-density lipoprotein (LDL) carries approximately 75% of total cholesterol (TC) that is carried by non-high density lipoprotein (HDL) particles (Jacobson et al., 2015). People with low LDL cholesterol (LDL-C) levels are less likely to develop CVD compared with those having average or high levels of LDL-C (Cohen et al., 2006; Ference et al., 2012). For instance, a long-term exposure to lower LDL-C is associated with a 54.5% reduction in the risk of coronary heart disease (CHD) for each mmol/L (38.7 mg/dl) reduction of LDL-C (Ference et al., 2012). The first line lipid-lowering drug, statins, can significantly reduce LDL-C levels, leading to a reduction in CVD events by 25%–40% (Michos et al., 2012; Silverman et al., 2016; Toth et al., 2019a). Moreover, the antibodies or siRNA of proprotein convertase subtilisin/kexin-type 9 (PCSK9) can further decrease LDL-C level as an add-on-statin therapy. However, patients-treated with statins in combination with PCSK9 inhibitors still experience ASCVD events even their LDL-C levels meet the clinical goals (Sampson et al., 2012; Nishikido and Ray, 2021; Su et al., 2021). Therefore, researchers are impelled to find novel strategies for treatment of residual ASCVD.

Given a low HDL cholesterol (HDL-C) level is a strong and independent risk factor associated with CVD events, enhancing HDL-C level has ever been expected to prevent and/or recover CVD (Catapano et al., 2016; Mach et al., 2020). Cholesteryl ester transport protein (CETP) inhibitors can significantly improve HDL-C levels and reverse cholesterol transport. However, these inhibitors are found to be useless in prevention of CVD events (Liu N et al., 2021). HDL dysfunction in patients with CVD may partially explain the failure of these CETP inhibitors (Sandesara et al., 2019). Recent studies suggested that although HDL-C is a useful risk biomarker, accumulating evidence from Mendelian randomization studies and other research have demonstrated that HDL-C is not a causal risk factor for ASCVD (Nordestgaard, 2016; Lincoff et al., 2017; Barter and Genest, 2019; Goyal et al., 2021). Therefore, researchers turn their attention to other targets for treatment of ASCVD.

Previously, whether the elevated plasma triglyceride (TG) levels are causally associated with ASCVD has been controversial. Genetic and epidemiological studies have demonstrated that TG and TG-rich lipoprotein (TGRL) are main causes of residual ASCVD (Do et al., 2013; Jørgensen et al., 2013; Thomsen et al., 2014; Nordestgaard, 2016; Generoso et al., 2019; Laufs et al., 2019; Matsunage et al., 2020; Farnier et al., 2021; Nordestgaard et al., 2021). For instance, approximately 26% adults in United State, including one-third of statin users, have a TG ≥ 150 mg/dl, and approximately 40% adults with diabetes have a TG ≥ 150 mg/dl despite statin use. These elevated TGs are associated with CVD risk even in patients with low LDL-C levels (Hoogeveen and Ballantyne, 2020; Toth et al., 2020). In the genome-wide association studies, the susceptibility sites for CHD are associated with genes involved in TG metabolism (Teslovich et al., 2010; Schunkert et al., 2011). Mendelian randomization studies also indicate that there is a causal relationship between TG metabolism and the risk of atherosclerosis (Johansen and Hegele, 2013; Jørgensen et al., 2013; Varbo et al., 2013; Thomsen et al., 2014; Si et al., 2021). The atherogenic effects of TG, TGRL, and TGRL metabolites are dependent on their roles in endothelial function, inflammation, oxidative stress, and formation of foam cells. In this review, we will make a short review of TG and TGRL metabolism, discuss the association between TG and ASCVD, summarize the atherogenic factors of TGRL, and outline the current advances in TG-lowering therapies and the targets with potential applications in TG modulation.

## A Short Review of TG and TGRL

TG is the major storage form of fatty acid (FA) within cells and in circulation (Alves-Bezerra and Cohen, 2017; Duran and Pradhan, 2021). Liver is the central organ for metabolism of FAs that are originated from the plasma and/or hepatocellular *de novo* biosynthesis. FA synthesis is precisely controlled by a series of enzymes including sterol regulatory element binding protein (SREBP) 1c. When glucose is abundant, plasma insulin activates the endoplasmic reticulum (ER) membrane-bound transcription factor SREBP-1c, which can upregulate genes related to FA biosynthesis (Alves-Bezerra and Cohen, 2017). Within hepatocytes, FA is esterified to glycerol-3-phosphate (G3P) to generate TG. It is estimated that more than 90% of the total TG is synthesized by the G3P pathway in most mammalians (Alves-Bezerra and Cohen, 2017; Lee and Ridgway, 2020). The acylation of G3P is a rate-limiting step because G3P acyltransferase (GPAT) family members have the lowest specific activity within the enzymes involved in TG synthesis. GPAT1 is highly expressed in the liver, and deficiency of GPAT1 can reduce the plasma level of TG and secretion rate of VLDL (Hammond et al., 2005; Neschen et al., 2005). TG synthesis pathways and its metabolism in liver have been well-documented in the literature (Alves-Bezerra and Cohen, 2017; Lee and Ridgway, 2020; Castillo-Núñez et al., 2022). The assembly of TG is the primary way for the liver to store and export FA. However, only a small amount of FA is stored in the form of TG as lipid droplet because most of the FAs are either oxidized in the mitochondrion or packaged in the core of very low-density lipoprotein (VLDL) as TG and secreted into the blood.

As TG is a kind of nonpolar and hydrophobic molecule, it must be combined with related proteins and lipids to form lipoprotein particles during transportation in blood (Do et al., 2013; Castillo-Núñez et al., 2022). In the liver, a large amount of TGs are assembled with cholesterol, phospholipids, and apolipoprotein (apo) B100 (apoB100) into VLDL. In the small intestine, dietary TG is decomposed into FA and monoglyceride or diglyceride before being absorbed by enterocytes. These decomposition products are reassembled into CM with cholesterol, phospholipids, and apoB48 (Julve et al., 2016; Santos-Baez and Ginsberg, 2020). Next, CMs are released into the lymphatic system and enter the circulation, where they obtain other apolipoproteins including apoCII, apoCIII, and apoE (Rosenson et al., 2014; Nakajima and Tanaka, 2018a). Of importance, microsomal triglyceride transfer protein (MTP) plays a key role in the assembly of VLDL and CM via transporting the related lipids to apoB particles (Iqbal et al., 2020). Furthermore, recent studies have demonstrated that CETP increases the production of VLDL-TG in response to estrogen treatment via enhancing the expression of nuclear receptor and small heterodimer partner in female CETP transgenic mice (Palmisano et al., 2016; Palmisano et al., 2021). Therefore, some endogenous molecules, such as estrogen, mediate sex-specific modulation of TG metabolism. The secreted VLDL and CM particles transport FAs to muscle and adipose tissue for energy usage and/or storage via the blood flow (Duran and Pradhan, 2021; Castillo-Núñez et al., 2022).

**FIGURE 1:**
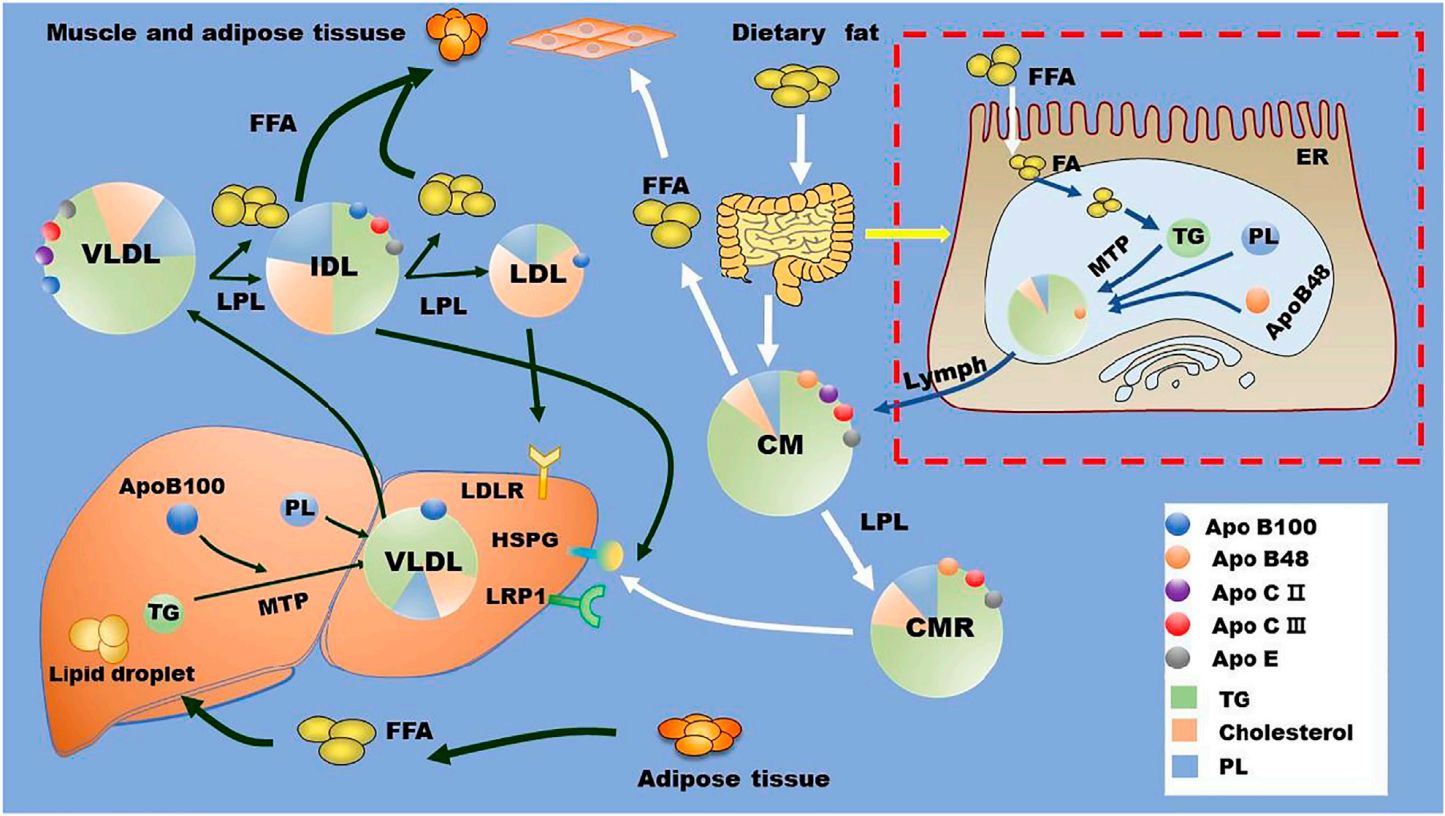
TG and TGRL metabolism. Dietary fat is metabolized by intestinal cells into FAs, which are reassembled with cholesterol, phospholipids, and apoB48 to form CMs. These CMs are released into blood *via* lymph. In the liver, exogenous and *de novo* synthesized FAs are assembled with cholesterol, phospholipids, and apoB100 to form VLDL with the assistant of MTP. In the circulation, LPL located at the surface of the capillary lumen hydrolyzes the TG in the core of TGRL (CM and VLDL) and promotes the production of TGRL remnants and free FAs. TGRL remnants are cleared by liver through receptors including HSPG, LDLR, LRP1, and other potentially unidentified receptors. Apo, apolipoprotein; CM, chylomicron; CMR, chylomicron residual; ER, endoplasmic reticulum; FA, fatty acid; FFA, free fatty acid; HSPG, heparan sulfate proteoglycan; IDL, intermediate density lipoprotein; LDL, low-density lipoprotein; LDLR, low-density lipoprotein receptor; LPL, lipoprotein lipase; LRP1, LDLR-related protein 1; MTP, microsomal triglyceride transfer protein; PL, phospholipids; TG, triglycerides; VLDL, very low-density lipoproteins.

In circulation, lipoprotein lipase (LPL) located at the surface of capillary lumen hydrolyzes TGs that are encapsulated in the core of CM and VLDL into FAs. LPL binds to its endothelial coenzyme, glycosylphosphatidylinositol-anchored HDL binding protein 1 (GPIHBP1), to provide a platform for lipolysis of apoB-containing lipoproteins on the surface of vascular endothelium (Davies et al., 2010; Young et al., 2019; Basu and Goldberg, 2020). Of note, the activity of LPL is highly regulated by several proteins, such as apoCII, apoCIII, apoE, and angiopoietin-like protein (ANGPTL)3, ANGPTL4, and ANGPTL8 (Rosenson et al., 2014; Wu et al., 2021). Along with TG hydrolysis, CM gradually turns into smaller and cholesterol-rich CM remnant (CMR), and VLDL becomes intermediate density lipoprotein (IDL), which is further catabolized to be LDL via hepatic TG lipase (HTGL) on the surface of hepatic sinusoidal endothelial cell cavity (Young and Zechner, 2013). TGRL remnants in circulation are cleared by liver receptors including LDL receptor (LDLR), LDLR-related protein (LRP)-1, scavenger receptor B type 1 (SR-B1), and heparan sulfate proteoglycan (HSPG) (Toth, 2016; Kockx and Kritharides, 2018; Christopoulou et al., 2019). For instance, small particles, such as LDL, are cleared through the binding of apoE and/or apoB to LDLR, while larger particles, such as CMR, are eliminated by the binding of apoE to HSPG and other potentially undefined hepatic receptors (Kockx and Kritharides, 2018; Christopoulou et al., 2019). The metabolism of TG and TGRL is summarized in Figure 1.

## Hypertriglyceridemia and Atherosclerosis

Generally, lipid analysis (such as TC and TG levels) is performed using overnight fasted blood samples. However, recent studies have demonstrated that non-fasting and fasting plasma samples show similar lipid profiles, and all these data can be used for prediction of CVD risk (Bansal et al., 2007; Nordestgaard et al., 2007; Jørgensen et al., 2013; Thomsen et al., 2014). On average, non-fasting plasma TG levels are approximately 0.3 mmol/L (27 mg/dl) higher than the corresponding fasting samples (Catapano et al., 2016; Mach et al., 2020). TG levels reach peak at 4–6 h after food intake. Therefore, people are in a state of non-fasting at most of the times within a day, and non-fasting lipid levels are more representative than those of fasting lipid profiles. Presently, the standard measurement of plasma TG is still performed under fasting conditions. Generally, the fasting TG concentration <1.7 mmol/L (150 mg/dl) is defined as normal, 1.7–11.4 mmol/L (150–1000 mg/dl) is defined as moderate HTG, and >11.4 mmol/L (1000 mg/dl) is defined as severe HTG (Parhofer and Laufs, 2019). Extreme HTG is rare and is defined as fasting TG concentration >20 mmol/L (∼1750 mg/dl) (Parhofer and Laufs, 2019; Mach et al., 2020). Of note, severe HTG is generally associated with pancreatitis (Laufs et al., 2019; Parhofer and Laufs, 2019). Although the threshold TG level of 1.7 mmol/L is accepted by all medical societies, moderate and severe HTG are differently defined by distinct medical societies. Such as, severe HTG is also defined as TG level >10 mmol/L (850 mg/dl) (Laufs et al., 2019).

Genetic studies indicate that HTG can be caused by both single gene and multiple gene variants (Dron and Hegele, 2020; Matsunage et al., 2020; Ginsberg et al., 2021; Tokgözoğlu and Libby, 2022). For instance, homozygous or biallelic variants in LPL, apoCII, apoCIII, apoAV, lipase maturation factor 1, GPIHBP1, and ANGPTLs are demonstrated to be correlated with HTG (Jørgensen et al., 2013; Rosenson et al., 2014; Dron and Hegele, 2020; Gill et al., 2021). TGRL is consisted of a TG, cholesterol ester, and cholesterol core that is surrounded by phospholipids and apolipoproteins. These apolipoproteins play important roles in CM assembly and degradation. Carriers of the rare non-synonymous mutation of apoAV have higher levels of plasma TG compared with those of non-carriers (Do et al., 2015). An E40K loss-of-function variant in the gene encoding ANGPTL4 is associated with substantially reduced plasma levels of TG in white persons (Folsom et al., 2008). Furthermore, GPIHBP1 deficiency develops severe plasma CM in mouse even on a low-fat diet (Beigneux et al., 2007). In apoA-IV knockout mice, larger CM particles are formed and the clearance of these larger CMs is significantly delayed in circulation compared with those of wild-type mice (Kohan et al., 2012). Mechanistically, apoA-IV may influence particle assembly and/or lipidation in the ER, thereby modulating CM size and secretion (Black, 2007). Furthermore, obese adolescents show higher levels of ANGPTL3 and apoCIII, which potentially inhibit LPL activity, leading to increased TGRL levels and residual atherosclerosis risk (Rodríguez-Mortera et al., 2020).

HTG is reported to affect 15–20% of the adult population and is associated with overweight, metabolic syndrome, and diabetes mellitus (Parhofer and Laufs, 2019). Of note, approximately 50% of patients with type 2 diabetes are accompanied with HTG (Parhofer and Laufs, 2019). Patients with mild to moderate HTG have a higher risk of atherosclerosis than people with normal TG (Crea, 2021; Nordestgaard et al., 2021; Tokgözoğlu and Libby, 2022). One study indicates that TG ≥ 150 and TG ≥ 200–499 mg/dl may enhance CVD risk by 25.0% and 34.9%, respectively (Toth et al., 2021). In patients with severe HTG, individuals with CMRs that are rich in TG also have an increased risk of atherosclerosis (Dron and Hegele, 2020). Accumulating epidemiological studies have indicated that plasma level of TG (both fasting and non-fasting) has a strong correlation with atherosclerosis, and elevated TG levels are an independent risk factor for ASCVD (Nordestgaard et al., 2007; Pirillo et al., 2014; Werner et al., 2014; Kockx and Kritharides, 2018; Shahid et al., 2018; Laufs et al., 2019). Some research support that non-fasting TG levels are more closely associated with incident CVD events than fasting TG levels (Bansal et al., 2007; Adiels et al., 2012). Of importance, each reduction of 88.5 mg/dl of TG level is associated with approximately 50% reduction in CVD risk (Jun et al., 2010). Therefore, lowering TG treatment can reduce the risk of ASCVD as that of lowering LDL-C (Ference et al., 2019). In 2019, European Society of Cardiology and European Atherosclerosis Society has clearly pointed out that TG ≥ 175 mg/dl is a risk factor of ASCVD events and TG-lowering therapy is recommended for residual ASCVD therapy (Mach et al., 2020).

## TGRL and Atherosclerosis

**FIGURE 2:**
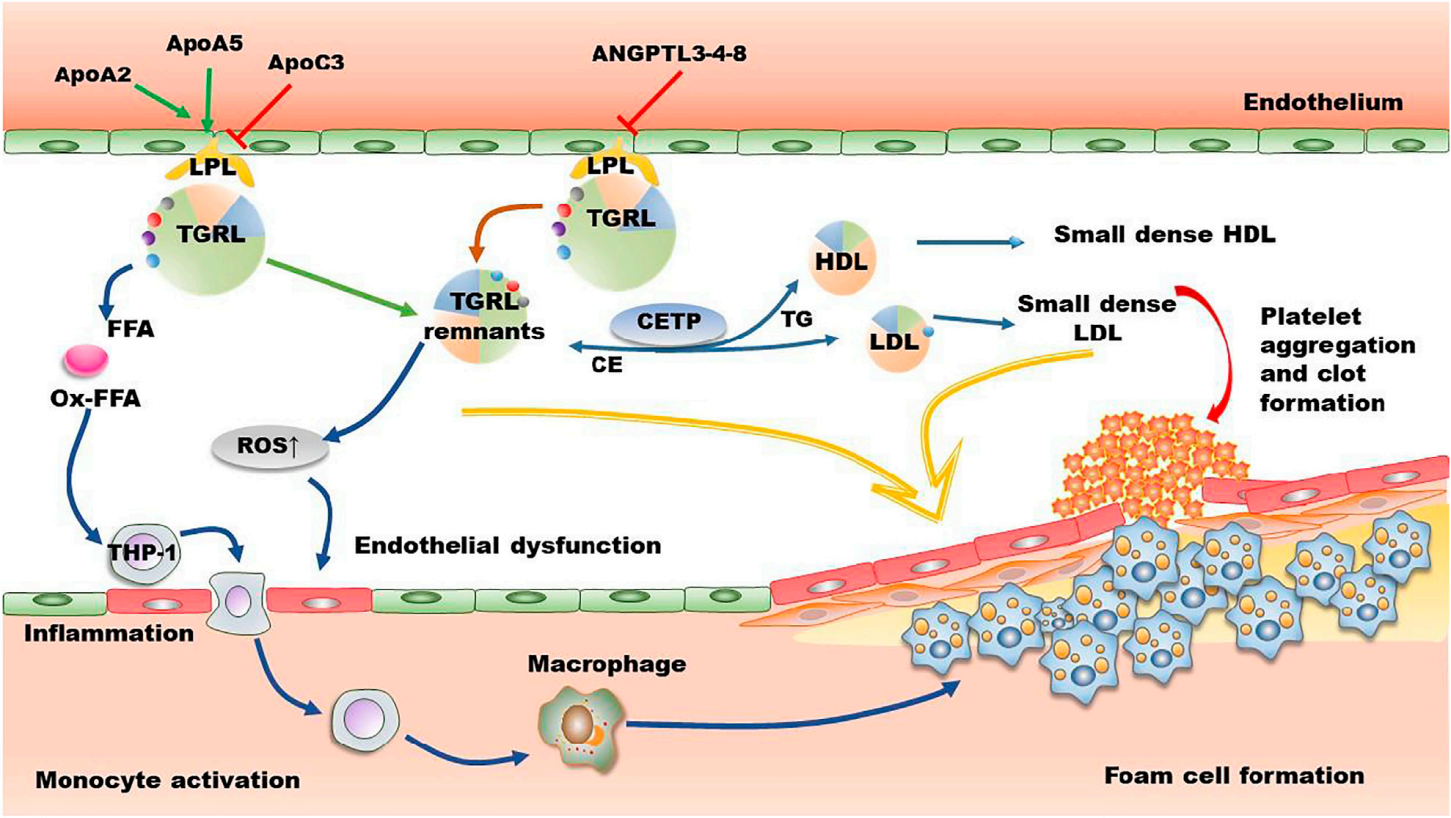
The mechanisms of TGRL on promoting atherosclerosis. In circulation, apoAII and apoAV enhance the activity of LPL, while apoAIII and ANGPTL3-4-8 suppress LPL-induced TGRL lipolysis. However, several studies demonstrate that apoAIII has no effect on the activity of LPL. The released FFA and the lipolysis process promote the production of ROS and secretion of inflammatory factors, leading to endothelial dysfunction and formation of foam cells. CETP-mediated lipid exchange between TGRL and HDL/LDL increases the production of small dense LDL and small dense HDL, further promoting deterioration of atherosclerosis. Furthermore, lipolytic products activate platelet and induce clot formation. ANGPTL, angiopoietin-like protein; Apo, apolipoprotein; CE, cholesteryl ester; CETP, cholesteryl ester transport protein; FFA, free fatty acid; HDL, high density lipoprotein; LDL, low-density lipoprotein; LPL, lipoprotein lipase; Ox-FFA, oxidized FFA; ROS, reactive oxygen species; TG, triglycerides; TGRL, triglyceride-rich lipoprotein.

TG is the main component of TGRL (CM and VLDL), and their remnants CMR and IDL (Duran and Pradhan, 2021; Castillo-Núñez et al., 2022). Therefore, plasma TG concentration is a biomarker for TGRL and their remnants in circulation. The methods used for isolation and quantification of TGRL remnants have been recently reviewed by distinct groups (Hoogeveen and Ballantyne, 2020; Duran and Pradhan, 2021). After meals, TGs are transported from the small intestine to bloodstream by CM particles, where they are converted to atherogenic CMRs by LPL in tissues (Ginsberg et al., 2021). Similarly, liver secreted VLDL particles are converted to IDL and then LDL by LPL and HTGL in circulation. TGRL and the hydrolyzed residuals including free FAs bind to leukocytes and endothelial cells in circulation, leading to a state of acute activation that is characterized by expression of integrins, generation of ROS, production of cytokines as well as a complement activation (DeVries et al., 2014). Recent studies have demonstrated that TGRL and their remnants are positively associated with atherosclerosis by up-regulating inflammation, oxidative stress, and foam cell formation as shown in Figure 2.

### TGRLs Activate Inflammation

Atherosclerosis is characterized as a chronic inflammatory disease. Of note, each mmol/L (39 mg/dl) increase of TGRL cholesterol is associated with a 37% increase of C-reactive protein level, suggesting TGRL cholesterol increases inflammatory response (Varbo et al., 2013). Furthermore, plasma levels of interleukin (IL)-6 and tumor necrosis factor-alpha (TNFα) are significantly higher in postprandial subjects than those in fasting state, suggesting that elevated levels of postprandial TGRL cholesterol are associated with inflammatory response, causing increased susceptibility for premature atherosclerosis (Twickler et al., 2003). TGRLs with high TG content up-regulate the level of TNF-α, thereby inducing the expression of vascular cell adhesion molecule (VCAM)-1 in human aortic endothelial cells and monocyte adhesion. On the contrary, TGRLs with low TG content have an atheroprotective effect by reducing VCAM-1 expression and monocyte recruitment (Gower et al., 2011; Wang et al., 2011; Sun et al., 2012). Postprandially released VLDL particles have an increased level of apoCIII (Wang et al., 2011; Sun et al., 2012), and these particles activate inflammation in endothelial cells by enhancing the protein kinase C (PKC)/NF-κB signaling pathway (Libby, 2007). Similarly, VLDL particles promote inflammation by activating the NF-κB signaling pathway in endothelial cells (Dichtl et al., 1999; den Hartigh et al., 2014). However, ApoCIII A allele at rs2070667 shows an inhibitory effect on polyunsaturated fatty acids (PUFA)-containing TGs and hepatic inflammation in nonalcoholic fatty liver disease (Xu et al., 2020).

Macrophage is a central link between lipid metabolism and inflammatory response. TG synthesis (lipid droplet formation) enhances macrophage inflammation (Castoldi et al., 2020). *In vitro*, VLDL enhances the expression of TNF-α, IL-1β, monocyte chemoattractant protein 1 (MCP-1), intercellular adhesion molecule-1 (ICAM-1), matrix metalloproteinase 3, and macrophage inflammatory protein 1-α. Mechanistically, VLDL activates mitogen-activated protein kinase (MAPK) signaling cascades including the phosphorylation of extracellular signal-regulated kinase (ERK) 1/2, c-Jun NH2-terminal kinase (JNK), and p38 MAPK (Jinno et al., 2011). VLDL particles further enhance the expression of TNF-α in macrophages that are induced by LPS *via* activating ERK1/2, MAPK kinase (MEK)1/2, and the transcription factor AP-1 rather than nuclear factor-κB (NF-κB) or peroxisome proliferator activated receptor (PPAR) γ (Stollenwerk et al., 2004). TGRL also induces inflammation by activating the inflammasome nucleotide binding domain like receptor family pyrrole domain containing protein 1 (NLRP1) (Bleda et al., 2016; Bleda et al., 2017). Furthermore, VLDL intensifies its pro-inflammatory effects by binding to LRP and activating the downstream p38 MAPK/NF-κB signaling pathway (Libby, 2007). Of note, ER stress and the unfolded protein response are also involved in TGRL-induced inflammation (Ozcan et al., 2004; Civelek et al., 2009; Wang et al., 2013). In addition, CMRs stimulate the expression of IL-1β via activating caspase-1 and NF-κB in THP-1 cells (Okumura et al., 2006). CMRs also activate human monocytes and enhance their migration *in vitro*, contributing to an inflammatory environment in the early stage of atherosclerosis (Bentley et al., 2011). The inflammation-associated hormone, growth and differentiation factor 15, is also involved in TGRL-mediated inflammation (Luan et al., 2019).

**FIGURE 3:**
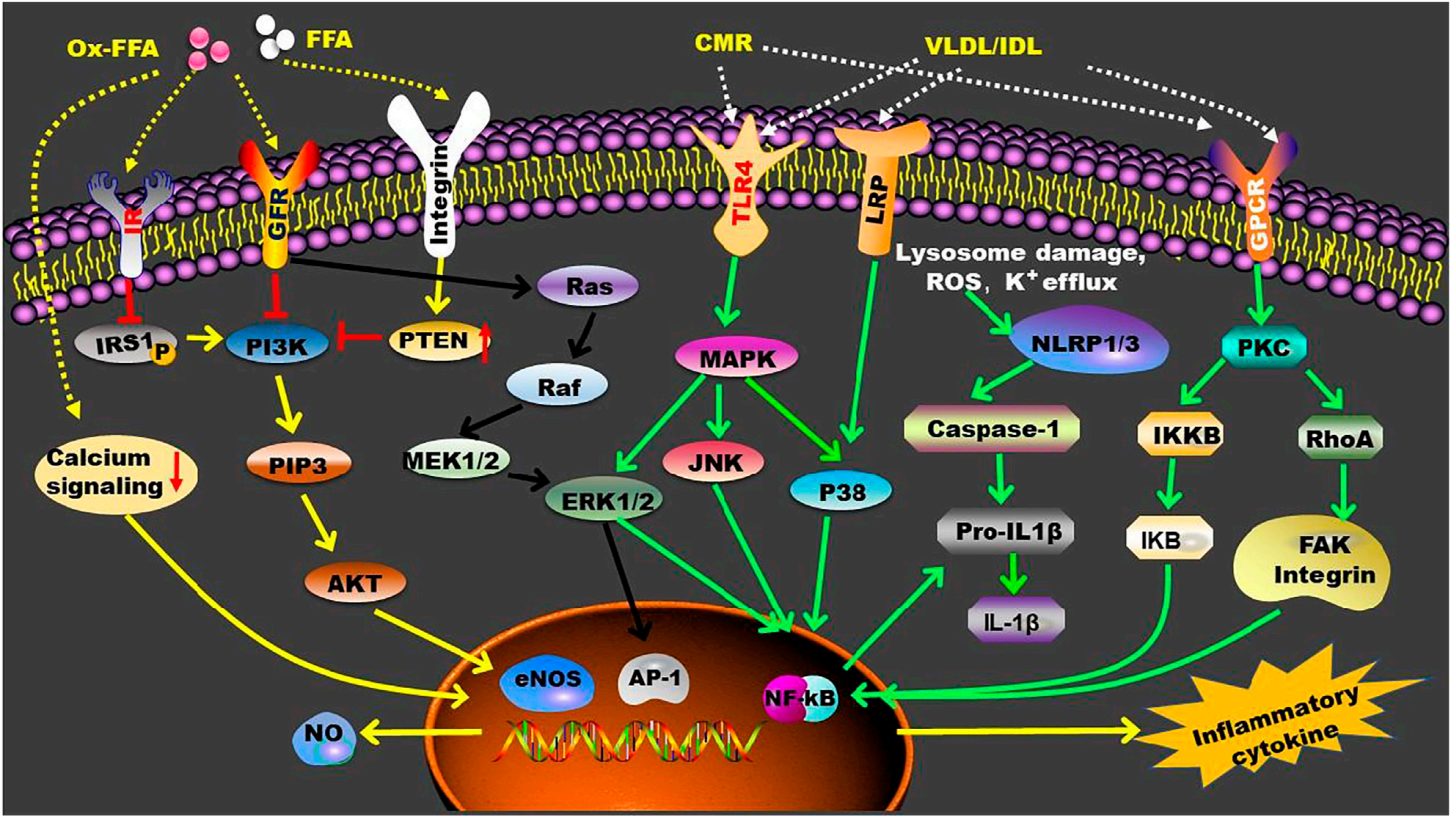
TGRL and TGRL metabolites-mediated inflammatory signaling pathways. In circulation, LPL converts TGRL to TGRL remnants, such as CMR and IDL, and promotes the production of FFA. These TGRL metabolites enhance inflammation by activating multiple receptors that located on the cell membrane. Furthermore, FFA can penetrate cell membrane and exert their functions intracellularly. AP-1, activating protein-1; eNOS, endothelial nitric oxide synthase; ERK1/2, extracellular signal-regulated kinase 1/2; FAK, focal adhesion kinase; FFA, free fatty acid; GFR, growth factor receptor; GPCR, G protein-coupled receptor; IKB, nuclear factor-kappa B inhibitor; IKKB, Inhibitor of kappa B kinase; IL-1β, interleukin-1β; IR, insulin receptor; IRS1, insulin receptor substrate 1; JNK, c-Jun NH2-terminal kinase; LRP, LDL receptor-related protein; MAPK, mitogen-activated protein kinase; MEK1/2, mitogen-activated protein kinase kinases 1/2; NF-kB, nuclear factor-kappa B; NLRP, nucleotide binding domain like receptor family pyrrole domain containing protein; NO, Nitric oxide; P38, P38 mitogen-activated protein kinase; PI3K, phosphoinositide 3-kinase; PIP3, phosphatidylinositol 3,4,5-trisphosphate; PKC, protein kinase C; PTEN, phosphatase and tensin homolog; RhoA, Ras homolog family member A; TRL-4, Toll-like receptor 4.

Accumulating evidence have demonstrated that TGRLs and their remnants increase endothelial inflammation and facilitate monocytes infiltration of the arterial wall. A previous study demonstrated that TGRL induces monocyte adhesion to vascular endothelial cells by sequentially activating the expression of PKC, RhoA, focal adhesion kinase, and integrins *in vitro*, suggesting a mechanism of TGRL remnants-mediated vascular inflammation during atherogenesis (Kawakami et al., 2002). TGRL remnants also induce the expression of TNF-α, VCAM-1, ICAM-1, E-selectin, and MCP-1 through modulation of lectin-like receptor for oxidized LDL (LOX-1) receptor and NF-κB-dependent nuclear transcription (Park et al., 2005). Furthermore, JNK and activating transcription factor 3 (ATF3) are involved in TGRL lipolysis products-induced vascular inflammation via upregulating the levels of IL-8 and E-selectin (Aung et al., 2013). Postprandial TGRL also stimulate inflammation via interferon regulatory factor-1, especially under shear stress in cultured endothelium (Sherrod DeVerse, et al., 2013). Oxylipids in TGRLs are found to promote endothelial inflammation following a high fat meal (Rajamani et al., 2019). Of importance, TGRL hydrolysis and the accumulation of intracellular TGs and free FAs, especially oxidized free FAs, play key roles in TGRL-mediated inflammation. Reductions in susceptibility of VLDL to LPL can attenuate the above inflammatory reactions (Saraswathi and Hasty, 2006; Jinno et al., 2011; Castillo-Núñez et al., 2022). FAs are transported into cells passively or actively by transporters including plasma membrane fatty acid-binding protein, fatty acid transport proteins, and cluster of differentiation 36 (Mallick and Duttaroy, 2022). Neutral and oxidized free FAs released during TGRL hydrolysis are found to induce endothelial inflammation and vascular apoptosis (Wang et al., 2009). The relationship between free FAs and inflammation has been reviewed recently by distinct groups (Mallick and Duttaroy, 2022; Panda et al., 2022; Ren et al., 2022). Some presently known inflammatory signaling pathways that are modulated by TGRL, TGRL remnants, and free FAs are summarized in Figure 3.

### TGRLs Induce Oxidative Stress and Endothelial Cell Dysfunction

Oxidative stress plays an important role in the progression of atherosclerosis by interacting with inflammation, foam cell formation, and endothelial dysfunction. TGRL remnant cholesterol stimulates NAD(P)H oxidase–dependent superoxide formation and cytokine secretion in human umbilical vein endothelial cells by activation of LOX-1, leading to reduction in cell viability (Shin et al., 2004). Furthermore, TGRL remnant cholesterol upregulates endothelial expression of ICAM-1, VCAM-1, and tissue factor through a redox-sensitive mechanism, thus affecting the progression of atherosclerosis (Doi et al., 2000). CMRs also cause rapid and prolonged generation of ROS in monocytes, thereby influencing monocyte activation and migration (Bentley et al., 2011). Exposure of J774 macrophages to a pro-oxidizing state further promotes CMR-induced accumulation of intracellular lipids (Napolitano et al., 2001).

Endothelial dysfunction is one of the early pathological mechanisms of atherosclerosis. Free FAs released during lipolysis of TGRL can activate NADPH oxidase- and cytochrome P450-mediated ROS production within endothelial cells, causing oxidative stress and dysfunction of endothelial barrier (Wang et al., 2009). The mechanisms of action of FAs on modulation of endothelium function have been reviewed recently by Mallick and Duttaroy (2022). Furthermore, high levels of TGRL remnant cholesterol and TG have a strong correlation with endothelial vasomotor dysfunction. These TGRL remnants increase the susceptibility of coronary endothelium to oxidative stress, leading to inhibition of nitric oxide-mediated vascular dilation (Nakamura et al., 2005). Of note, postprandial elevated TGRL remnant cholesterol is associated with atherosclerotic progression even in normolipidemic subjects by affecting endothelial dysfunction, such as endothelium-dependent vasorelaxation (Inoue et al., 1998; Funada et al., 2002). TGRL lipolysis induces ROS production and alterations of lipid raft in morphology and protein components, such as LRP, nitric oxide synthase, and caveolin-1, leading to endothelial dysfunction (Wang et al., 2008). Furthermore, TGRL and their lipolytic products activate platelet aggregation and clot formation by suppressing fibrinolysis and promoting the assembly of prothombinase complex, the expression of plasminogen activator inhibitor-1 and its antigen, and the endothelial expression of tissue factor (Gianturco and Bradley, 1999; Toth, 2016; Reiner, 2017). Furthermore, TGs are found to increase the risk of atherosclerosis by increasing plasma viscosity, leading to impaired microcirculatory flow and enhanced interactions between atherogenic lipoproteins and endothelium (Rosenson et al., 2001).

### TGRLs Promote Foam Cell Formation

Lipid accumulation in the subcutaneous space of endothelium is a key characteristic of atherosclerosis (Lin et al., 2021). Reductions in plasma TG levels are associated with reduced all-cause mortality in CVD patients potentially due to the low cholesterol content in TGRL and TGRL remnant particles. Indeed, TGRL remnant cholesterol is an independent risk factor for menopausal women with CHD (Feng X. et al., 2020). Another study indicates that the cholesterol levels of TGRL are a residual risk of future CVDs in patients with stable CHD and even in those with LDL-C < 70 mg/dl after statin therapy (Fujihara et al., 2019). Of note, the elevated postprandial VLDL residues are the main cause of the occurrence and development of atherosclerosis (Nakajima and Tanaka, 2018b). Furthermore, the reduced TGRL removal efficacy maybe a causal factor of the increased atherosclerosis in elderly people (Maranhão et al., 2020).

TGRLs carry approximately 25% of the TC carried by non-HDL particles. Due to the big size, the cholesterol content carried by each TGRL particle is approximately 5–20 times higher than that carried by each LDL particle (Toth, 2016; Ginsberg et al., 2021). Except for CM particles, TGRL and TGRL remnant particles, can penetrate blood vessel and be recognized and directly ingested by macrophages through the ligand apoE located at the surface of TGRL (Ohmura, 2019; Ginsberg et al., 2021). As is known, only modified LDL (such as oxidation) particles are ingested by macrophages. TGRLs are the only natural, unmodified lipoproteins that can cause rapid lipid accumulation in macrophages as revealed *in vitro* (Gianturco and Bradley, 1999; Peng and Wu, 2022). First, TGRLs are trapped in artery walls through the interaction between positively charged residues on apoB with negatively charged groups of proteoglycans located at the endothelium lining (Sandesara et al., 2019). In the subcutaneous space, TGRL particles are internalized by macrophages and smooth muscle cells, contributing to plaque formation and development (Padro et al., 2021). Therefore, TGRL is more pathogenic than LDL in causing atherosclerosis.

Indeed, both apoB48 and apoB100 are found in human aortic atherosclerotic plaques, indicating that TGRLs are involved in the formation of atherosclerotic plaque (Nakano et al., 2008; Behbodikhah et al., 2021). Furthermore, the majority of apoB proteins isolated from human atherosclerotic plaques are derived from VLDL and IDL, but not LDL, suggesting that VLDL and IDL play a key atherosclerotic role in the intima of the arteries (Rapp et al., 1994; Proctor and Mamo, 1998; Castillo-Núñez et al., 2022). Furthermore, a proportion of arterial plaque cholesterol is derived from VLDL and its residue IDL in patients with mild-to-moderate HTG (Gill et al., 2021). Of note, plasma VLDL cholesterol (VLDL-C) is an increased risk factor for major adverse CVD events, independent of the established risk factors such as LDL-C (Heidemann et al., 2021). It seems that the remnant cholesterol, but not TG, in TGRL particles is a causal factor of atherosclerosis (Jørgensen et al., 2013; Varbo and Nordestgaard, 2016; Dron and Hegele, 2020). However, TGs in TGRL particles assist the ingestion process of the cells, such as macrophages, involved in plaque foam cell formation. Unlike VLDL particles isolated from people with normal TG levels, VLDL particles obtained from patients with HTG have a high affinity for scavenger receptors, such as LDLR and VLDLR, that are specifically expressed by monocytes, macrophages, and endothelial cells (Gianturco et al., 1982; Takahashi, 2017).

CMRs also contribute to lipid accumulation in the atherosclerotic plaques. Unlike LDL particles, CMRs carrying dietary lipids induce the formation of foam cells without the oxidation process in circulation, and the uptake of CMRs by macrophages can induce intracellular accumulation of both TG and cholesterol. The rate of uptake and lipid accumulation is affected by the type of dietary fat in the granules (Botham et al., 2007; Morita, 2016). In the absence of LDLR or its ligand apoE, CMRs still contribute to lipid accumulation during atherosclerotic plaque formation (Fujioka et al., 1998; Bravo and Napolitano, 2007). For instance, apoB48 receptor is involved in CMR uptake and foam cell formation independent of the apoE-mediated pathway. Of note, apoB48 receptor can be used for further uptake of TGRL particles even when macrophages are accumulated with a large amount of lipids (Kawakami et al., 2005; Bermudez et al., 2012). In macrophages, CMR internalization results in rapid accumulation of cholesterol in lysosomes and cell death due to lysosomal instability, thereby promoting atherosclerosis deterioration (Wakita et al., 2015). Additionally, TG contained in TGRL particles can activate CETP, which promotes the exchange of core lipids between major lipoproteins, promoting the accumulation of residual cholesterol in TGRL remnants, thereby aggravating atherosclerosis (Brinton, 2015). For instance, CETP mediates the exchange of TG in TGRL with cholesterol in LDL, thereby promoting the production of small, dense LDL particles that are more atherosclerotic than LDL particles (Gianturco et al., 1982; Merkel, 2009). Similarly, TGRL promotes HDL remodeling and the formation of smaller, low-cholesterol HDL particles that are lack of atherosclerotic protection (Feng M. et al., 2020).

## Strategies for Treatment of Hypertriglyceridemia

Except for statins, clinical drugs that can be used to treat HTG are fibrates, niacin, and omega-3 fatty acids (Preston Mason, 2019). Several previous reviews have summarized the effects of these clinically used drugs for TG-lowering (Sando and Knight, 2015; Simons, 2018; Feingold, 2021). Furthermore, our team demonstrated that exogenous supplement of N-acetylneuraminic acid can reduce TG by more than 60% in apoE^*(−/−)*^ mice (Guo et al., 2016; Hou et al., 2019). Here, we make a short review about these clinically used drugs, and then focus on some targets with potential applications for TG-lowering.

### Clinical Drugs

#### Statins

Statins are the standard therapy for many types of dyslipidemias because they can effectively decrease the endogenous biosynthesis of cholesterol via inhibiting 3-hydroxy-3-methyl glutaryl coenzyme A reductase and enhance the hepatic uptake of LDL particles by up-regulating LDLR through SREBP-2. In individuals with normal TG levels, statins have little effects on plasma VLDL. However, statins decrease VLDL and CM and their remnants via improving hepatic clearance in patients with HTG (Caslake and Packard, 2004). A previous study demonstrates that statin treatment decreases not only fasting TG but also postprandial TGs (Mora-Rodriguez et al., 2020). However, a survey in United State adults (9593 participants) indicates that the prevalence of TG < 150, 150–199, and ≥200 mg/dl is 75.3%, 12.8%, and 11.9% in adults without statin treatment; among statin users, the ratios are 68.4%, 16.2%, and 15.4%, respectively. Furthermore, the estimated mean 10-years ASCVD risk from TG < 150 to ≥500 mg/dl, ranges from 11.3% to 19.1% in statin users and 6.0%–15.6% in nonusers. These data suggest that statin treatment moderately elevates TG levels as well as ASCVD risk in patients with TG > 150 mg/dl (Fan et al., 2019). Another meta-analysis of randomized-controlled trials demonstrates that statins only reduce plasma TG by 8.4% in children and adolescents with familial hypercholesterolaemia (Anagnostis et al., 2020). Patients with increased CVD risk generally display high levels of plasma TGs and low levels of HDL-C, even after statin therapy (Larsson et al., 2014). Of importance, statin-treated patients with TG levels ≥ 150 mg/dl have even worse CVD risk than those with TG < 150 mg/dl (Toth et al., 2019a; Toth et al., 2019b). Collectively, statins show limited effects on TG-lowering in patients with dyslipidemia.

#### Fibrates

Mechanistically, fibrates exert their bioactivity primarily by activating PPARα (Chapman et al., 2010). Fibrate treatment reduces plasma TG levels by approximately 20%–70% (Katsiki et al., 2013; Wang et al., 2015). Compared with placebo, gemfibrozil (1.2 g/d) reduces TG level by 31% and increases HDL-C by 6% along with a reduction in the risk of CVD events by 4.4% in CHD patients (Rubins et al., 1999). Oral bezafibrate treatment (400 mg/d, twice) leads to a significant reduction in serum TG level (42.7%) as well as increases in HDL2-C, HDL3-C, and plasma content and activity of LPL. Bezafibrate may increase HDL3-C and HDL2-C by promoting TGRL catabolism and the conversion of HDL3 to HDL2, respectively (Sakuma et al., 2003). Fenofibrate or fenofibric acid can significantly reduce TG level in combination with statin (Huang et al., 2009; Ouwens et al., 2015). A meta-analysis demonstrates that fibrate reduces TG level by 46.5% in combination with statin. However, this combination increases risk of side effects (Choi et al., 2014). Pemafibrate (k-877), a selective PPARα modulator, is found to be superior to fenofibrate (106.6 mg/d) in TG lowering in patients with high TG (≥1.7 mmol/L and <5.7 mmol/L) and relatively low HDL-C levels at the dosage of 0.2–0.4 mg/d (Ishibashi et al., 2018). However, a meta-analysis suggests that pemafibrate reduces plasma TG levels and increases HDL-C similar as that of fenofibrate (Ida et al., 2019). In a multicenter, randomized, double-blind, phase IV study, fenofibrate significantly decreases TG levels from 269.8 to 145.5 mg/dl as an add-on-statin therapy, while statin monotherapy has no effect on TG levels (Park et al., 2021). A recent retrospective longitudinal study indicates that pemafibrate significantly reduces plasma TG by 43.8% and increases HDL-C by 10.8% in patients with dyslipidemia after 3 months treatment. Furthermore, this molecule improves liver function and serum levels of uric acid and hemoglobin A1c (Yanai et al., 2022).

#### Niacin

Niacin (nicotinic acid) decreases TG levels by up to 30%, and reduces LDL-C and lipoprotein (α)) levels to the greatest extent by 15% and 30%, respectively (Chapman et al., 2010). It inhibits the lipolysis of adipose tissue and reduces the flow of free FAs to the liver, leading to a reduction in hepatic synthesis of VLDL (Khetarpal et al., 2016). This molecule can successfully reduce TGs in patients with familial CM syndrome potentially by reducing the production of apoB48 and CM (Pang et al., 2016). Furthermore, niacin mimics the role of the physiological ligand β-hydroxybutyrate by interacting with the type 3 hydroxycarboxylic acid receptor (Chaudhry et al., 2018). In a clinical follow-up study, niacin treatment decreases TG level from 164 mg/dl to 122 mg/dl, reduces LDL-C from 74 mg/dl to 62 mg/dl, and increases HDL-C level from 35 mg/dl to 42 mg/dl. However, there is no incremental clinical benefit from the addition of niacin (1.5–2 g/d) to statin treatment (40–80 mg/d) during the 3 years of follow-up period in patients with ASCVD (Boden et al., 2011). A large randomized trial demonstrates that although the extended-release niacin treatment reduces TG levels by 33 mg/dl on average compared with placebo, the addition of niacin to statin therapy has no effect on reducing the risk of major vascular events. On the contrary, niacin is found to increase the risk of myopathy, especially in patient after simvastatin treatment (HPS2-THRIVE Collaborative Group, 2013). Therefore, the use of niacin in clinical practice is limited due to its useless for reducing CVD events and the high incidence of adverse reactions.

#### Omega-3 Fatty Acid

The beneficial roles and metabolism of PUFA have been recently review by several groups (Chen et al., 2022; Mallick and Duttaroy, 2022; Ren et al., 2022). Omega 3 FAs belong to the family of PUFA and play an important role in the formation and stability of cell membranes as well as cell physiology (Ganda et al., 2018). Omega-3 FAs, such as eicosapentaenoic acid (EPA) and docosahexaenoic acid (DHA), can reduce TG levels, improve blood vessel function, and suppress inflammation, platelet aggregation, liver steatosis, and insulin resistance (Mozaffarian and Wu, 2011; Shahidi and Ambigaipalan, 2018). A previous review indicates that DHA and EPA treatment is associated with a net decrease in TG by 22.4% and 15.6%, respectively. Furthermore, DHA supplement is associated with more significant increases in LDL-C and HDL-C compared to that of EPA (Jacobson et al., 2012; Kotwal et al., 2012). These Omega 3 FAs affect lipid raft organization by disrupting acyl chain packing and molecular order within lipid rafts, thereby modulating protein lateral distribution and signaling (Shaikh, 2012). Of note, EPA and DHA have different effects on membrane bilayer width, membrane fluidity, and cholesterol crystalline domain formation, suggesting omegar-3 FAs with different structural characteristics may show distinct effects (Preston Mason et al., 2016). The TG-lowering mechanisms of action of these compounds are associated with increased FA degradation through peroxisome β-oxidation, reduced hepatic fat production, and enhanced TG clearance in circulation (Harris et al., 2008).

In a 12-weeks clinical trial, the EPA ethyl ester, AMR101, reduces TG level by 10.1% and 21.5% at the dosage of 2 and 4 g/d, respectively, in high-risk statin-treated patients with residually high TG (>200 and <500 mg/dl) (Ballantyne et al., 2012). In patients with fasting TG levels of 1.52–5.63 mmol/L and LDL-C levels of 1.06–2.59 mmol/L, 2 g/d of EPA ethyl (twice daily) significantly reduces the relative risk of ischemic events by 25% compared with the placebo group (Bhatt et al., 2019). Of note, EPA (1.8 g/d) combined with pitavastatin (4 mg/d) is found to reduce coronary plaque volume and reinforce plaque stabilization compared to pitavastatin monotherapy (Watanabe et al., 2017). Another study in Japan demonstrates that EPA (1.8 g/d) reduces CVD events by 19% in patients receiving statin therapy and decreases CVD events by 53% in patients with TG ≥ 150 mg/dl and HDL-C < 40 mg/dl, suggesting EPA is more effective in patient with abnormal TG and HDL-C levels (Saito et al., 2008). Icosapent ethyl is recommended for treatment of ASCVD patients with fasting TG between 139 and 499 mg/dl in combination with statin therapy (Orringer et al., 2019). However, omega-3 FA supplementation has no protective effects in patients with diabetes without evidence of CVD (Bowman et al., 2018). A meta-analysis involving 77917 high-risk individuals suggests that omegar-3 FAs have no significant association with fatal and nonfatal CHD or any major vascular events (Aung et al., 2018). Epanova, a mix of omega-3 free FAs, lowers plasma TG level by up to 31%. In 2018, the STRENGTH study was designed to check whether 4 g/day of epanova can reduce the incidence of CVD events in patients with HTG and low levels of HDL-C (Nicholls et al., 2018). This STRENGTH study terminated on 8 January 2020 demonstrates that there is no significant difference between omega-3 FA treatment and corn oil intervention (Nicholls et al., 2020; Nissen et al., 2021; Reyes-Soffer, 2021). Therefore, omega-3 FAs mix may also have limited application in clinical therapy for HTG.

## Emerging Targets for Lowering Triglyceride

Mendelian randomization and genetic studies provide evidence of potential therapeutic targets for reducing TG and the risk of ASCVD. These potential targets include LPL and LPL-related proteins, such as apoCIII, apoCII, apoAV, ANGPTL4, and GPIHBP1, which cause alterations in TG levels and are related to the development of ASCVD. For instance, in a systematic review and meta-analysis, apoCIII is found to cause HTG and atherosclerosis (Wyler von Ballmoos et al., 2015; Rocha et al., 2017). The proatherosclerotic effects of GPIHBP1 deficiency are probably caused by the markedly elevated levels of CM/VLDL, which exacerbate atherosclerosis through increasing the formation of TGRL remnants and generation of proatherogenic lipid products (Vallerie and Bornfeldt, 2015). Furthermore, accumulating evidence have demonstrated that gut microbiota is also associated with TG metabolism.

### Targeting LPL

LPL maintains TG homeostasis in blood and is the rate-limiting enzyme for the hydrolysis of TGs that are encapsulated in the core of TGRL particles (Wang and Eckel, 2009; Tada et al., 2018; Kumari et al., 2021). It has been reported that gain-of-function and loss-of-function gene mutations of LPL lead to the imbalance of plasma TG levels, thereby influencing CVD events (Rip et al., 2006; Wang and Eckel, 2009; Burnett et al., 2019). LPL S447X is a naturally occurring gain-of-function mutation (Rip et al., 2006). In 2012, alipogene tiparvovec (AAV1-LPL^S447X^) was approved in Europe for treatment of severe HTG and recurrent pancreatitis in patients with complete loss-of-function of LPL. This highly active recombinant LPL with S447X variant reduces the fasting TG level by >40% in half of the patients between 3 and 12 weeks (Gaudet et al., 2013). Furthermore, several interesting compounds are found to increase the activity of LPL. Among these agonists, 50F10 is found to stabilize LPL *in vitro* and successfully reduce postprandial HTG in apoAV^*(−/−)*^ mice (Larsson et al., 2014). The agonist NO-1886 (ibrolipim) mainly increases the mRNA level of LPL, while the compound C10d primarily affects the hydrolysis activity of LPL (Larsson et al., 2014). Although a long-term administration of NO-1886 significantly inhibits the development of coronary atherosclerosis, this compound shows severe side effects. Except for lowering TG, the agonist C10d lowers TC, body fat, and fatty liver, suggesting its potential application in HTG treatment (Geldenhuys et al., 2017).

### Targeting ApoCIII

ApoCIII is synthesized in the liver and intestine, and it is distributed in TGRL and HDL particles (Borén et al., 2020). This apolipoprotein is previously demonstrated to be an effective inhibitor of LPL (Christopoulou et al., 2019). However, several studies have shown that apoCIII has pleiotropic effects in regulating the metabolism of TGRL without affecting LPL (Gordts et al., 2016; Kovrov et al., 2022). ApoCIII mainly suppresses the hepatic clearance of TGRLs and their remnants through LDLR and LRP-1, thereby inducing HTG (Gordts et al., 2016; Christopoulou et al., 2019; Kegulian et al., 2019; Reyes-Soffer and Ginsberg, 2019). People with apoCIII loss-of-function mutations are associated with approximately 40% reductions in plasma TG and the risk of CVD (Pollin et al., 2008; Crosby et al., 2014; Jørgensen et al., 2014). Therefore, apoCIII is a therapeutic target for patients with severe HTG. Volanesorsen is an antisense oligonucleotide targeting apoCIII mRNA, and it is developed for treatment of familial CM syndrome (FCS), HTG, and familial partial lipodystrophy (Gouni-Berthold, 2017; Paik and Duggan, 2019). Based on the beneficial effects observed in the phase III study, volanesorsen was approved in the European Union for treatment of adult FCS patients in May 2019 (Hegele et al., 2018; Corbin et al., 2020; Gouni-Berthold et al., 2021; Lazarte and Hegele, 2021). The average TG level in FCS patients decreases by 77% after treatment using volanesorsen (300 mg) once a week for 3 months (Witztum et al., 2019). Subcutaneous injection of volanesorsen (300 mg) once a week for 3 months reduces the average TG level by 71.2% in patients that have applied conventional TG-lowering therapy but have a fasting TG > 500 mg/dl (Gouni-Berthold et al., 2021). However, this compound may cause side effects such as thrombocytopenia (Witztum et al., 2019).

### Targeting ApoCII

ApoCII is a key cofactor for activation of LPL. A complete deficiency of apoCII causes the same phenotype, severe HTG, as LPL deficiency (Hegele et al., 2020). The apoCII mimetic peptide (C-II-a, 30 mg/kg) is found to reduce plasma TG level by 85% in apoE^*(−/−)*^ mice (Amar et al., 2015). Intravenous injection of this short peptide (0.2–5 μmol/L) reverses HTG in apoCII^*(−/−)*^ mice in a dose-dependent manner (Sakurai et al., 2016). C-II-a peptide is found to promote plasma clearance of TG-rich lipid emulsions and improve the following incorporation of FAs from these TG emulsions into specific peripheral tissues (Komatsu et al., 2019). However, this mimic peptide only acutely activates LPL due to its short half-life (1.33 h) (Sakurai et al., 2016). Recent studies have shown that apoCII mimic peptide D6PV is a novel compound for the treatment of HTG and apoCII deficiency (Wolska et al., 2020a; Wolska et al., 2020b). In apoCII^*(−/−)*^ mice and human apoCIII-transgenic mice, this peptide consisted of 40-amino acid causes a rapid decrease in plasma TG and apoB by approximately 80% and 65%, respectively. Furthermore, it also works independent of LPL (Wolska et al., 2020a). Of importance, D6PV displays good TG-lowering bioactivity in nonhuman primates and shows an extended terminal half-life of 42–50 h (Wolska et al., 2020a). These data suggest that apoCII mimetic peptides have an attractive future for treatment of HTG.

### Targeting ApoAⅤ

Genetic association studies have established a clear link between apoAⅤ variation and TG metabolism (Nilsson et al., 2011; Flores-Viveros et al., 2019). ApoAV variants affect not only TG concentration but also the distribution of lipoprotein subclasses, shifting them to atherogenic particles in high-risk subjects (Guardiola et al., 2015; Guardiola and Ribalta, 2017). A previous study demonstrates that three mutations including p.(Ser232_Leu235)del, p.Leu253Pro, and p.Asp332ValfsX4 are the direct cause of HTG by apoAⅤ (Mendoza-Barberá et al., 2013). Furthermore, there is a significant association between c.56C > G (rs3135506) apoAⅤ gene polymorphism and coronary artery disease in the Moroccan population (Morjane et al., 2020). Liver-derived apoAⅤ facilitates LPL-mediated TG hydrolysis in circulation. Furthermore, apoAⅤ is co-localized with perilipin through binding LRP-1, and it reduces intracellular TG concentration by suppressing adipogenesis-related factors in adipocytes (Su et al., 2020; Xu et al., 2020). ApoAV shows a protective effect against atherosclerosis in apoE2 gene knock-in and human apoAV transgenic mice via reducing TG and the residual particles rich in cholesterol esters, such as LDL and VLDL (Mansouri et al., 2008). Intravenous injection of wild-type apoAV reconstituted HDL significantly reduces TG by 60% in apoAV^*(−/−)*^ mice, and this effect requires the functional GPIHBP1-LPL-apoAV axis (Shu et al., 2010). Furthermore, adenovirus overexpression of human apoAV reduces serum levels of TG and cholesterol in mice (Van der Vliet et al., 2002). However, apoAV is involved in fructose-induced metabolic dysregulation and is associated with hepatic steatosis. Furthermore, there is a significantly lower levels of hepatic TG in apoAV^*(−/−)*^ mice compared with the control (Ress et al., 2020). Therefore, the working mechanisms of action of apoAV and the actual application for treatment of HTG by targeting apoAV need to be clarified in future.

### Targeting ANGPTL

ANGPTL is a family of secreted glycoproteins consisting of eight members (ANGPTL1-8). These proteins, especially ANGPTL3, ANGPTL4, and ANGPTL8 are found to regulate the activity of LPL (Li et al., 2020; Kumari et al., 2021). In the following, we mainly describe several intensively studied ANGPTL members that have potential applications for treatment of HTG.

### ANGPTL3

ANGPTL3 is a secreted protein mostly expressed in the liver. This protein increases the plasma levels of TG and LDL-C. However, loss-of-function variants in ANGPTL3 are associated with decreased plasma levels of TG, LDL-C, and HDL-C, as well as reduced ASCVD risk (Musunuru et al., 2012; Dewey et al., 2017). In mice, lack of ANGPTL3 reduces the plasma levels of TG, TC, and free FA, and increases the activity of LPL (Koishi et al., 2002; Fujimoto et al., 2006). In humans, evinacumab, an ANGPTL3 antibody, reduces fasting TG and LDL-C levels by approximately 76% and 23%, respectively, without affecting LDLR (Dewey et al., 2017). In an open-label study, 4 weeks evinacumab treatment reduces the plasma levels of TG, LDL-C, apoB, non-HDL-C, and HDL-C by 47%, 49%, 46%, 49%, and 36%, respectively, in patients with homozygous familial hypercholesterolemia (Gaudet et al., 2017). Evinacumab increases the fractional catabolic rate of IDL apoB and LDL apoB, suggesting this molecule may improve hepatic clearance of TGRL remnants (Reeskamp et al., 2021). The fully human monoclonal antibody (REGN1500) has a high affinity with ANGPTL3, and its effectiveness in reducing the plasma TG and LDL-C levels has been confirmed in monkeys and mice (Gusarova et al., 2015). Targeting ANGPTL3 mRNA by the antisense oligonucleotide, named as ANGPTL3-L_Rx_, causes reductions in the levels of ANGPTL3 protein by 46.6%–84.5%, TG by 33.2%–63.1%, LDL-Cs by 1.3%–32.9%, VLDL-C by 27.9%–60.0%, non-HDL-C by10.0%–36.6%, apoB by 3.4%–25.7%, and apoCIII by18.9%–58.8% in mice (Graham et al., 2017). Gene editing through CRISPR-Cas9 technology has been established as a potential strategy for treatment of patients with atherosclerotic dyslipidemia. For instance, injection of BE3-ANGPTL3 causes reductions in TG, TC, and ANGPTL3 by 49%, 51%, and 19%, respectively, in LDLR^*(−/−)*^ mice (Chadwick et al., 2018). Furthermore, a lipid nanoparticle delivery platform has recently been developed for targeted-delivery of CRISPR-Cas9-based genome editing of ANGPTL3. In this study, the reductions in ANGPTL3 mRNA and plasma TG level are stable for at least 100 days after a single administration (Qiu et al., 2021).

### ANGPTL4

ANGPTL4 is mainly expressed in liver, adipose tissue, kidney, intestine, and heart. This protein plays an important role in lipid metabolism, especially in TG metabolism (Aryal et al., 2019). It mediates fasting-induced repression of LPL activity by promoting LPL unfolding, thereby enhancing degradation of LPL. However, this protein may show distinct bioactivity in distinct organs or cells. The biological functions of ANGPTL4 have been previously reviewed by Aryal et al. (2019) and Kersten (2021). ANGPTL4 derived from liver and adipose tissue primarily acts as an endocrine factor that regulates systemic lipid metabolism, while ANGPTL4 in macrophages reduces the formation of foam cells (Yang et al., 2020). Like ANGPTL3, loss-of-function mutations in ANGPTL4 are associated with low TG levels and reduced CVD risk (Dewey et al., 2016). ANGPTL4 monoclonal antibody (REGN1001) inhibits ANGPTL4 and reduces plasma levels of TG in mice and non-human primates (Dewey et al., 2016). Compared to wild-type mice, ANGPTL4^*(−/−)*^ mice have lower levels of TG and TC due to the enhanced VLDL clearance and decreased VLDL production. Of note, the anti-ANGPTL4 monoclonal antibody, 14D12, is found to reduce TG by 50% in C57BL/6J mice. This antibody also reduces plasma levels of TG in LDLR^*(−/−)*^, apoE^*(−/−)*^, and db/db mice (Desai et al., 2007). Adipocyte-derived ANGPTL4 plays a key role in regulation of plasma TG in mice fed a regular chow diet, but this effect is diminished after a chronic high-fat diet feeding (Spitler et al., 2021). Of importance, clinical trials are needed for determining the actual effects of inhibiting ANGPTL4 on TG-lowering.

### ANGPTL 8

ANGPTL8, also known as adipin/betatrophin, regulates LPL activity in the heart and skeletal muscle. This protein is also expressed in liver and adipose tissue (Zhang, 2016). Although ANGPTL8 has a functional LPL inhibitory motif, it only inhibits LPL and increases plasma TG levels in the presence of ANGPTL3 or possibly other ANGPTL family members in mice (Haller et al., 2017). Therefore, ANGPTL8 seems to increase plasma TG level by interacting with ANGPTL3. The fully human monoclonal antibody, REGN3776, can bind monkey and human ANGPTL8 with a high affinity, and reduces plasma TG by up-regulating LPL activity in humanized ANGPTL8 mice. In addition, blocking ANGPTL8 by this antibody reduces serum TG and increases serum HDL-C in cynomolgus monkeys with spontaneous HTG (Gusarova et al., 2017). An ANGPTL3-4-8 model has been provided for explaining the mechanisms of action of ANGPTLs in regulation of TG metabolism (Zhang, 2016). In brief, food intake induces the expression of ANGPTL8 in the liver and white adipose tissue. In the liver, ANGPTL8 activates ANGPTL3 and promotes the formation of ANGPTL3-8 complexes, which finally suppress the activity of LPL in circulation. In the white adipose tissue, ANGPTL8 promotes the formation of ANGPTL4-8 complexes, which enhance the activity of LPL locally. On the contrary, fasting inactivates the expression of ANGPTL8, thereby modulating LPL by an inverse way (Zhang and Zhang, 2022).

### Other ANGPTLs

ANGPTL5 is primarily expressed in adipose tissue and heart. This protein is positively associated with obesity, type 2 diabetes, oxidized LDL, and especially glucose metabolism disorders, suggesting ANGPTL5 is involved in modulation of TG and glucose homeostasis (Alghanim et al., 2019; Hammad et al., 2020; Liu Y. Z et al., 2021). The plasma level of ANGPTL7 is also elevated in obese subjects and is positively associated with TG level, suggesting ANGPTL7 may be explored as a therapeutic target for modulating TG metabolism (Abu-Farha et al., 2017; Liu Y. Z et al., 2021). As reviewed previously, ANGPTL2 primarily derived from visceral fat is positively associated with inflammation and insulin resistance, while ANGPTL6 expressed in the liver is found to counteract obesity and insulin resistance by suppressing gluconeogenesis and enhancing energy expenditure (Kadomatsu et al., 2011). A recent study demonstrates that serum ANGPTL6 levels are a valuable predictor of metabolic syndrome (Namkung et al., 2019). Furthermore, ANGPTL6 is suggested to primarily maintain glucose homeostasis in response to hyperglycemia (Fan et al., 2020). Among the 8 ANGPTL members, ANGPTL1 shows a limited correlation with TG metabolism (Carbone et al., 2018). Although ANGPTL5-7 are associated with TG metabolism, the underlying mechanisms of action of these three ANGPTLs are still far from clear.

### Targeting Gut Microbiota

In recent years, the relationship between gut microbiota and lipid metabolism has been paid more and more attentions. Cotillard et al. point out that reductions of microbial abundance in obese patients are related to the elevated levels of serum TC and TG (Cotillard et al., 2013). Similarly, another study suggests that individuals with low microbial gene counts have higher TG levels than individuals with high microbial gene counts (Le Chatelier et al., 2013). Compared with healthy volunteers, the phylum *Bacteroides* is decreased and the order *Lactobacillus* is increased in patients with coronary artery disease (Emoto et al., 2016). In apoE^*(−/−)*^ mice, the relative abundances of *Verrucomicrobia*, *Bacteroidaceae*, *Bacteroides*, and *Akkermansia* are positively correlated with serum levels of TC, TG, HDL-C, and LDL-C. In addition, the relative abundance of Ruminococcaceae is positively correlated with HDL-C level, and the abundance of Rikenellaceae is negatively correlated with TG and LDL-C levels (Liu et al., 2020a).

Probiotics are live bacteria that colonize the gastrointestinal tract and endow beneficial effects for health. Some probiotics alleviate fat by modulating gut microbiota-short chain FA-hormone axis (Yadav et al., 2013). Of note, supplementation of *Lactobacillus plantarum* FRT10 is found to reduce the body weight, fat weight, and hepatic TG via upregulating the mRNA expression of PPARα and carnitine palmitoyltransferase-1α and down-regulating the mRNA expression of SREBP-1 and TG synthase diacylglycerol acyltransferase 1 in the liver of mice fed a high-fat diet (Cai et al., 2020). Furthermore, *Lactobacillus plantarum* FRT10 intervention is found to increase the abundance of *Lactobacillus*, *Bifidobacterium*, and *Akkermansia*, which could improve the imbalance of gut flora caused by a high-fat diet (Cai et al., 2020). Liraglutide is a glucagon-like peptide-1 (GLP-1) analog. This molecule significantly reduces hepatic TG content, insulin resistance, and serum LDL-C in db/db mice. Mechanistically, liraglutide significantly increases the abundance of *Akkermansia*, *Romboutsia*, and *norank_f_Bacteroidales_S24-7_group*, and decreases the abundance of *Klebsiella*, *Anaerotruncus*, *Bacteroides*, *Lachnospiraceae_UCG-001*, *Lachnospiraceae_NK4A136_group*, *Ruminiclostridium*, and *Desulfovibrio* (Liu et al., 2020b). Furthermore, oligofructose promotes satiety and reduces plasma TG in rats fed a high-fat diet by up-regulating the level of GLP-1 in the gut (Cani et al., 2005). Another study demonstrates that oligofructose increases the abundance of *Bifidobacteria* and *lactobacilli* in the gut of obese rats (Bomhof et al., 2014). Some polysaccharides may also exert their TG-lowering effects by regulating the gut microbiota (Yin et al., 2021; Li et al., 2022).

## Future Directions

Statins only provide 25%–40% reductions in CVD risk, and high TG levels are closely associated with residual CVD risk. Cholesterol carried by TGRLs and their remnants is a causal factor of ASCVD except for LDL-C. The structural characteristics of TGRLs and their remnants, such as lipid content, apolipoprotein components, particle size, and retention time in circulation, determine the burden of atherosclerosis (Aguilar Salinas and John Chapman, 2020; Packard et al., 2020; Duran and Pradhan, 2021). Although there are several available methods for quantification of TGRLs and their remnants, more specific methods are needed to accurately determine and quantify the subclasses of these particles that are derived from different metabolic pathways. The recently developed omics technologies may assist the clarification of the component of these particles. These data will clarify what kinds of TGRLs and their remnants primarily determine the progression of atherosclerosis. Furthermore, the underlying mechanisms of action of the recently identified therapeutic targets, such as ANGPTLs, on modulating TG metabolism and even their own metabolism or interactions between each other are still far from clear.

Of importance, the presently used clinical TG-lowering drugs, such as fibrate, omega-3 FAs, and niacin, show equivocal effects or even futile in reduction of CVD risk by monotherapy or in combination with statin. The reasons need to be clarified in future. Although several novel compounds exhibit good TG-lowering and/or even CVD protective effects in animal models and/or clinical trials, their actual functions need to be verified in practice. Furthermore, pill burden of the patients with dyslipidemia is another question need to be resolved (Bittner, 2019; Toth et al., 2019b). Last but not the least, lifestyle changes, including reductions in carbohydrate (such as glucose, sucrose, and starch) intake, alcohol intake, smoking, and body weight, are the main measures to control HTG.

## Glossary

ANGPTL: Angiopoietin-like protein
apo: Apolipoprotein
ASCVD: Atherosclerotic cardiovascular disease
ATF3: Activating transcription factor 3
CETP: Cholesteryl ester transport protein
CHD: Coronary heart disease
CM: Chylomicron
CMR: CM remnant
CVD: Cardiovascular disease
DHA: Docosahexaenoic acid
EPA: Eicosapentaenoic acid
ER: Endoplasmic reticulum
ERK: Extracellular signal-regulated kinase
FA: Fatty acid
FCS: Familial CM syndrome
G3P: Glycerol-3-phosphate
GLP-1: Glucagon-like peptide-1
GPAT: G3P acyltransferase
GPIHBP1: Glycosylphosphatidylinositol-anchored HDL binding protein 1
HDL: High density lipoprotein
HDL-C: HDL cholesterol
HSPG: Heparan sulfate proteoglycan
HTG: Hypertriglyceridemia
HTGL: Hepatic TG lipase
ICAM-1: Intercellular adhesion molecule-1
IDL: Intermediate density lipoprotein
IL: Interleukin
JNK: c-Jun NH2-terminal kinase
LDL: Low-density lipoprotein
LDL-C: LDL cholesterol
LDLR: LDL receptor
LOX-1: Lectin-like receptor for oxidized LDL
LPL: Lipoprotein lipase
LRP-1: LDLR-related protein 1
MAPK: Mitogen-activated protein kinase
MCP: Monocyte chemoattractant protein
MEK: MAPK kinase
MMP: Matrix metalloproteinase
MTP: Microsomal triglyceride transfer protein
NF-κB: Nuclear factor-κB
NLRP1: Nucleotide binding domain like receptor family pyrrole domain containing protein 1
PCSK9: Proprotein convertase subtilisin/kexin-type 9
PKC: Protein kinase C
PPAR: Peroxisome proliferator activated receptor
PUFA: Polyunsaturated fatty acids
SR-B1: Scavenger receptor B type 1
SREBP: Sterol regulatory element binding protein
TG: Triglyceride
TGRL: TG-rich lipoprotein
TNF-α: Tumor necrosis factor-alpha
VCAM: Vascular cell adhesion molecule
VLDL: Very low-density lipoprotein
VLDL-C: VLDL cholesterol
